# Cutaneous Metastasis With Squamous Differentiation in Triple-Negative Breast Cancer: A Rare Case of Histologic Transformation With Clinical Implications

**DOI:** 10.7759/cureus.83672

**Published:** 2025-05-07

**Authors:** Samar Patankar, Ameera Majeed

**Affiliations:** 1 School of Medicine, St. George's University, Saint George, GRD; 2 School of Medicine, Dubai Medical University, Dubai, ARE

**Keywords:** cutaneous metastasis of the breast cancer, diagnosis and treatment, incidence and prognosis, squamous metaplasia, triple-negative breast cancer

## Abstract

Breast cancer is a frequently encountered malignancy in females, with its incidence influenced by a variety of factors. Among its subtypes, triple-negative breast cancer (TNBC) tends to have a more aggressive clinical course and limited treatment options. Cutaneous metastasis with squamous differentiation is an uncommon manifestation typically associated with advanced or recurrent disease. We report the case of a 63-year-old female previously diagnosed and treated for TNBC in Syria, who later presented to our institution with advanced disease. Despite further management efforts, the disease progressed rapidly, and the patient ultimately passed away. This case highlights the aggressive nature of certain breast cancer subtypes and the importance of recognizing atypical metastatic presentations.

## Introduction

Triple-negative breast cancer (TNBC) is an aggressive subtype of breast cancer that lacks estrogen, progesterone, and human epidermal growth factor receptor 2 (HER2) and is responsible for more than 15-20% of all breast cancers [1]. The lack of these receptors is determined by immunohistochemistry (IHC), and HER2 negativity is confirmed by fluorescence in situ hybridization when IHC is equivocal. It is typically observed in young African American women and Hispanic women who carry a mutation in the BRCA1 gene and is associated with higher rates of recurrence and mortality, especially in the early years following diagnosis [2]. Cutaneous metastasis (CM) is a recognized complication of breast cancer, but its frequency varies by subtype. In a retrospective analysis conducted by Kong et al., it was found that skin metastasis was present in 42.4% of hormone receptor-positive breast cancer cases versus 23.2% of TNBC cases [3]. Though TNBC-associated CM is less common, it tends to appear earlier in the disease course and is often associated with more aggressive disease and a poorer prognosis. This case report highlights the aggressive nature of CM in TNBC and intends to create awareness about the prognosis of this disease.

## Case presentation

We present a case of a 63-year-old female from Syria with a known history of recurrent metastatic TNBC initially diagnosed in Syria in October 2023, with skin and pulmonary metastasis diagnosed in July 2024. She had received neoadjuvant chemotherapy of doxorubicin and cyclophosphamide for four cycles, followed by carboplatin/paclitaxel for six cycles from October 2023 till April 2024. In May 2024, she underwent a bilateral mastectomy and right axillary lymph node dissection, where postoperative histopathology confirmed a triple-negative subtype malignancy. She received one cycle of capecitabine.

**Figure 1:**
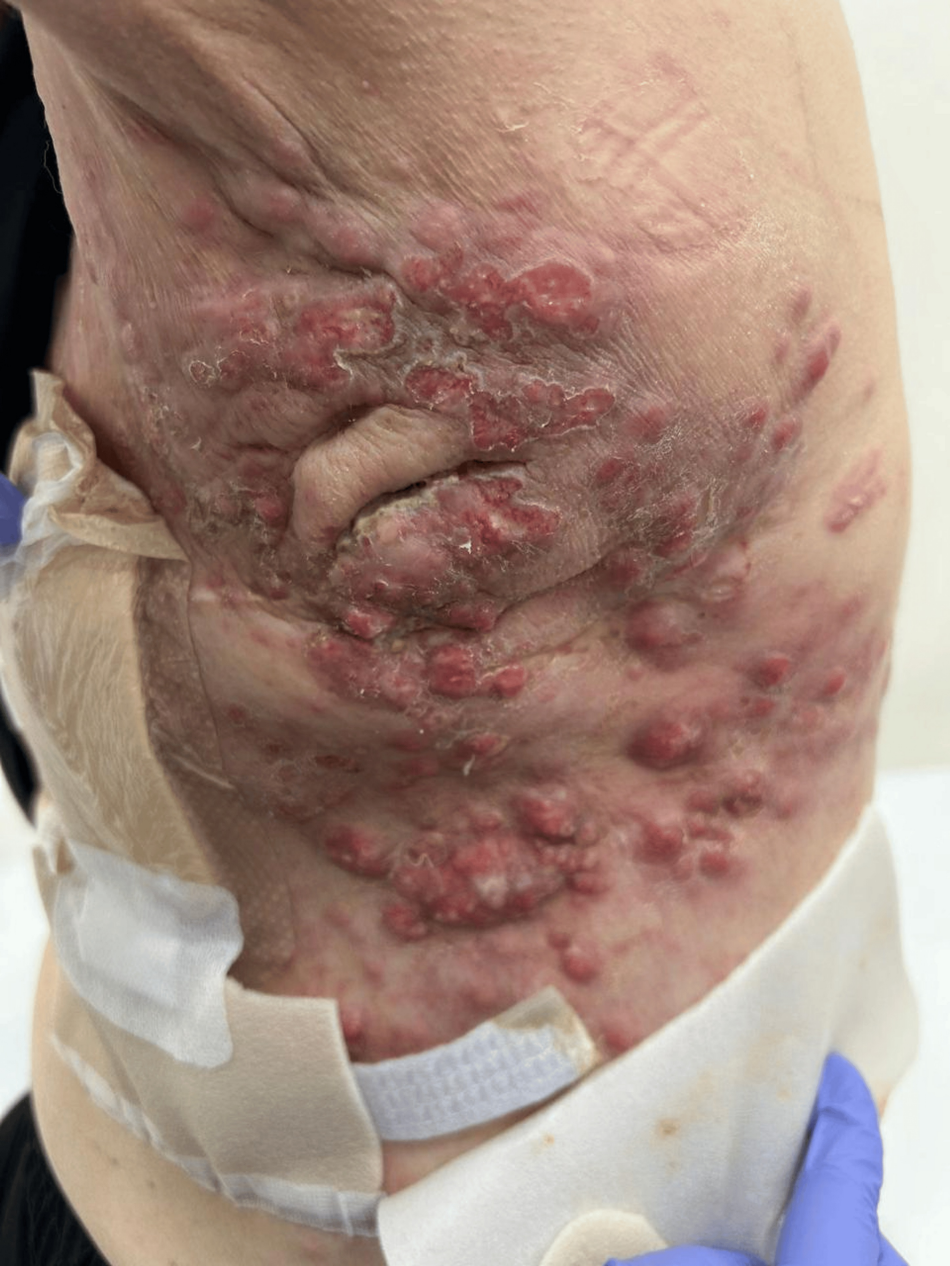
CM in TNBC This image is taken from the patient's left posterior chest wall. It shows multiple erythematous, firm nodules of varying sizes, some coalescing into larger irregular patches. There is evidence of significant crusting, scaling, and ulceration with hemorrhagic exudate. CM: cutaneous metastasis, TNBC: triple-negative breast cancer

**Figure 2:**
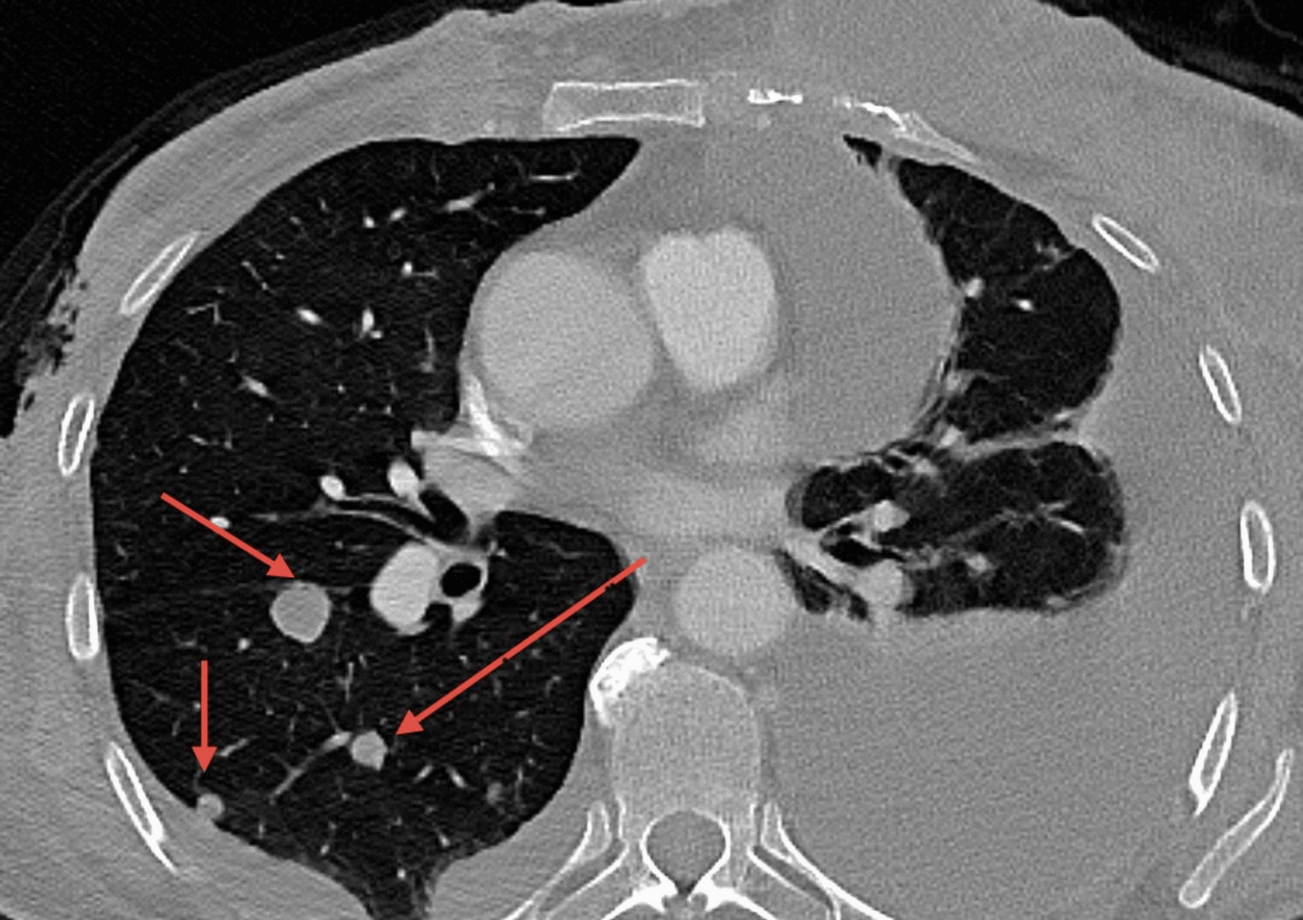
Axial CT scan of the chest showing pulmonary metastasis This axial chest CT shows numerous hypodense lung nodules of varying sizes, spread across various areas of the lung. CT: computed tomography

**Figure 3:**
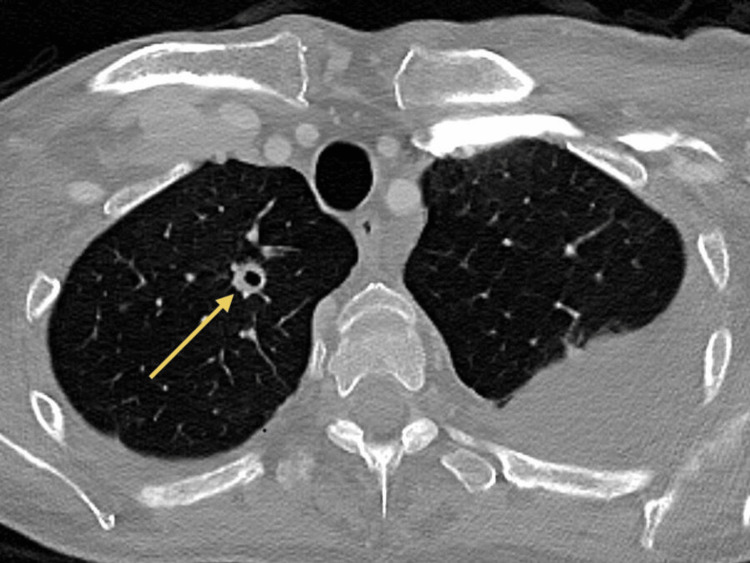
Axial CT scan of the chest showing pulmonary metastasis In this axial CT, central cavitation is present in certain nodules in the right apical segment. CT: computed tomography

**Figure 4:**
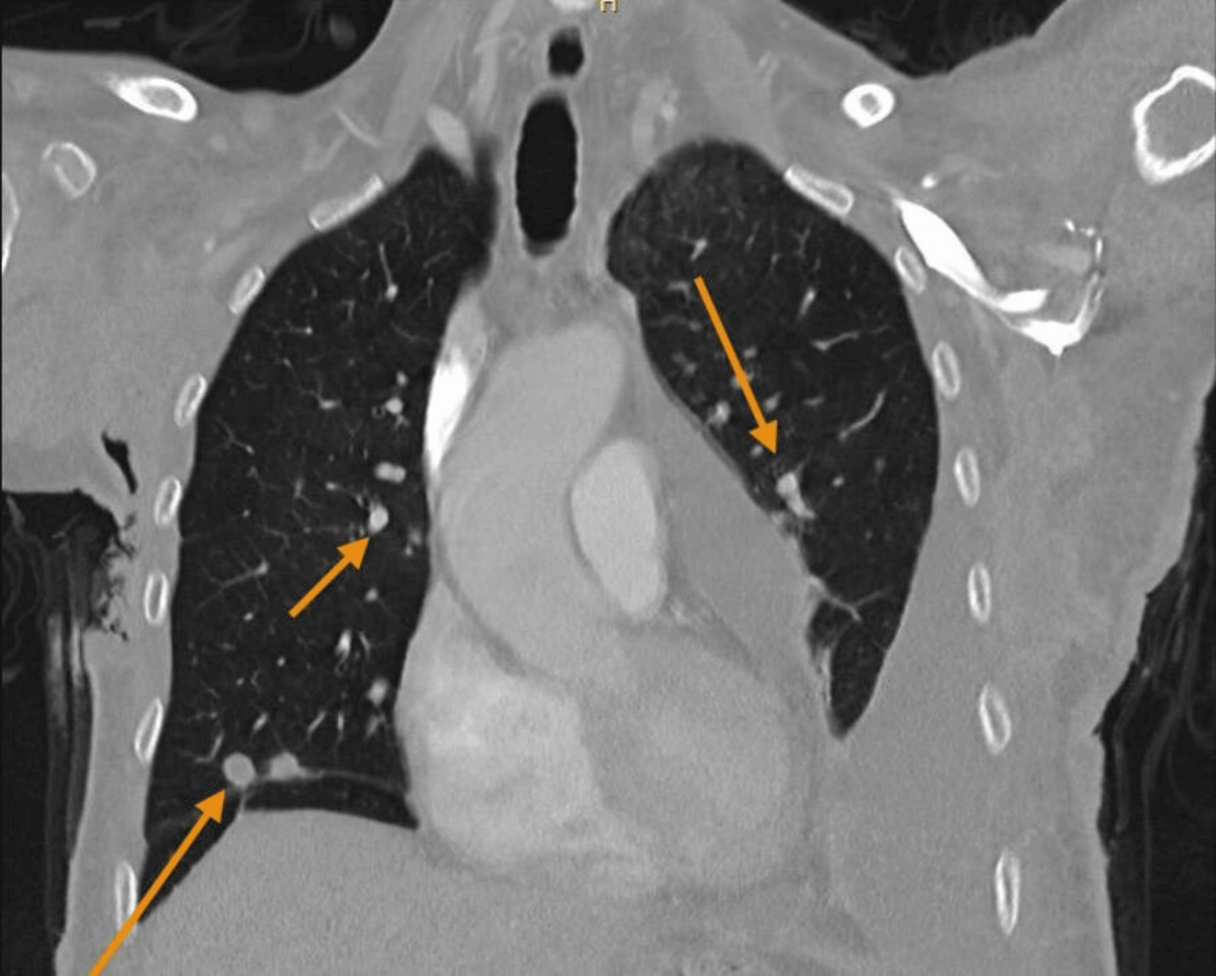
Coronal CT scan of the chest showing pulmonary metastasis CT: computed tomography

In July 2024, she developed new skin nodules (Figure 1). She underwent a chest wall biopsy, and the reports indicated poorly differentiated carcinoma with squamous metaplasia (images not available as the procedure was performed in Syria). She was treated with 15 fractions of radiotherapy. Systemic therapy was resumed with capecitabine and vinorelbine from September 2024 to November 2024. Later, CT of the chest, abdomen, and pelvis was done to assess for disease progression, which was positive for multiple pulmonary nodules (Figures 2-4). She was started on two cycles of gemcitabine in December 2024.

She presented to the emergency department in January 2025 with a wound infection associated with high-grade fever and foul-smelling, dark purulent discharge from her wound on the right chest wall. Inflammatory markers were elevated, and cultures were positive for *Klebsiella oxytoca*, *Staphylococcus aureus*, and *Acinetobacter baumannii*. She was admitted under the care of general surgery, where she received IV antibiotics and wound care. Cultures and lab investigations were repeated after two days, which indicated significant clinical improvement. She was discharged after five days of her admission with a dose of oral amoxicillin-clavulanic acid and an outpatient wound care plan. Additionally, she continued with the third cycle of gemcitabine.

**Figure 5:**
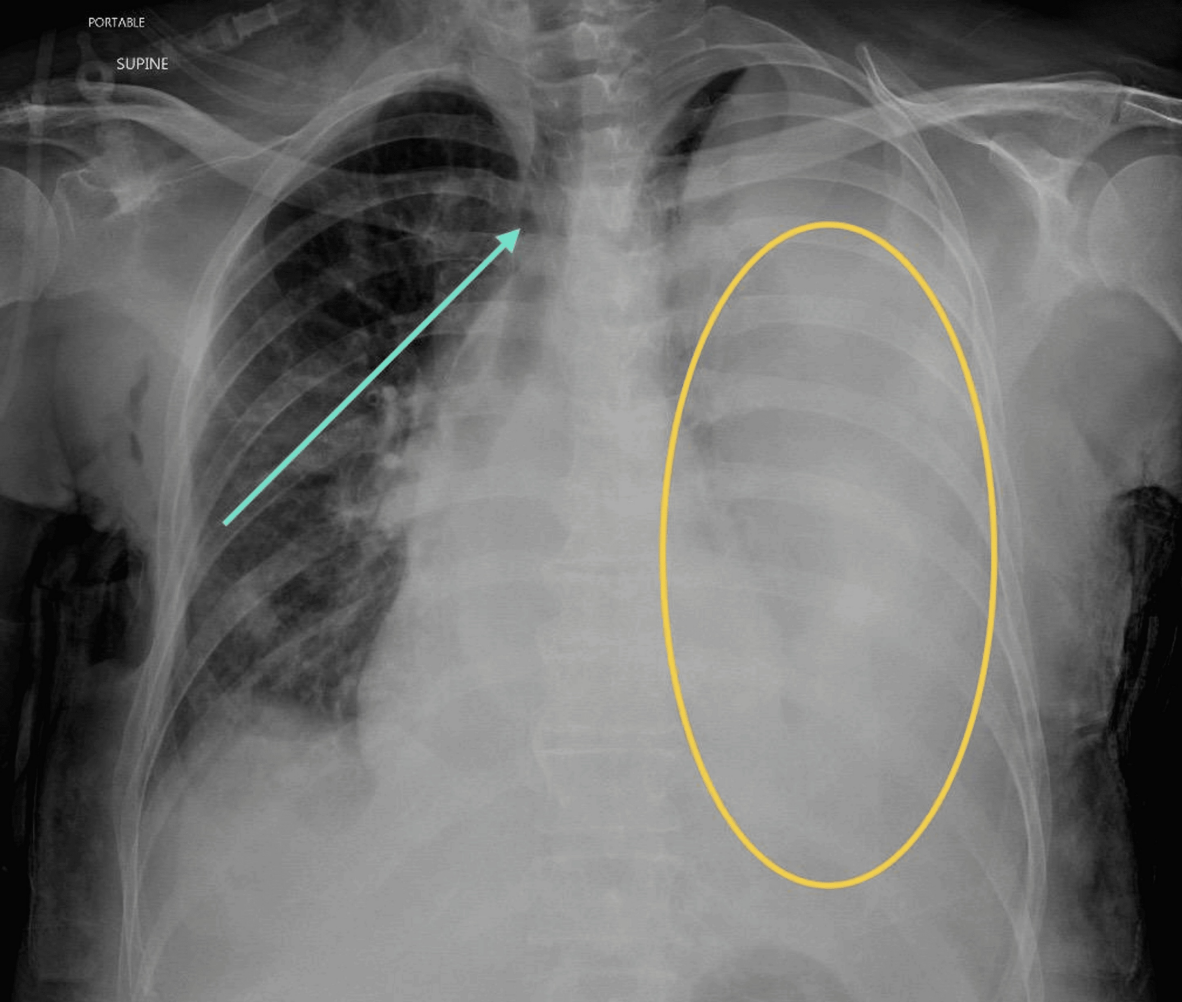
AP view of the chest X-ray showing left-sided pleural effusion The patient is in the supine position. This AP chest X-ray depicts a large left-sided pleural effusion (yellow circle) with rightward mediastinal shift (blue arrow). AP: anteroposterior

**Figure 6:**
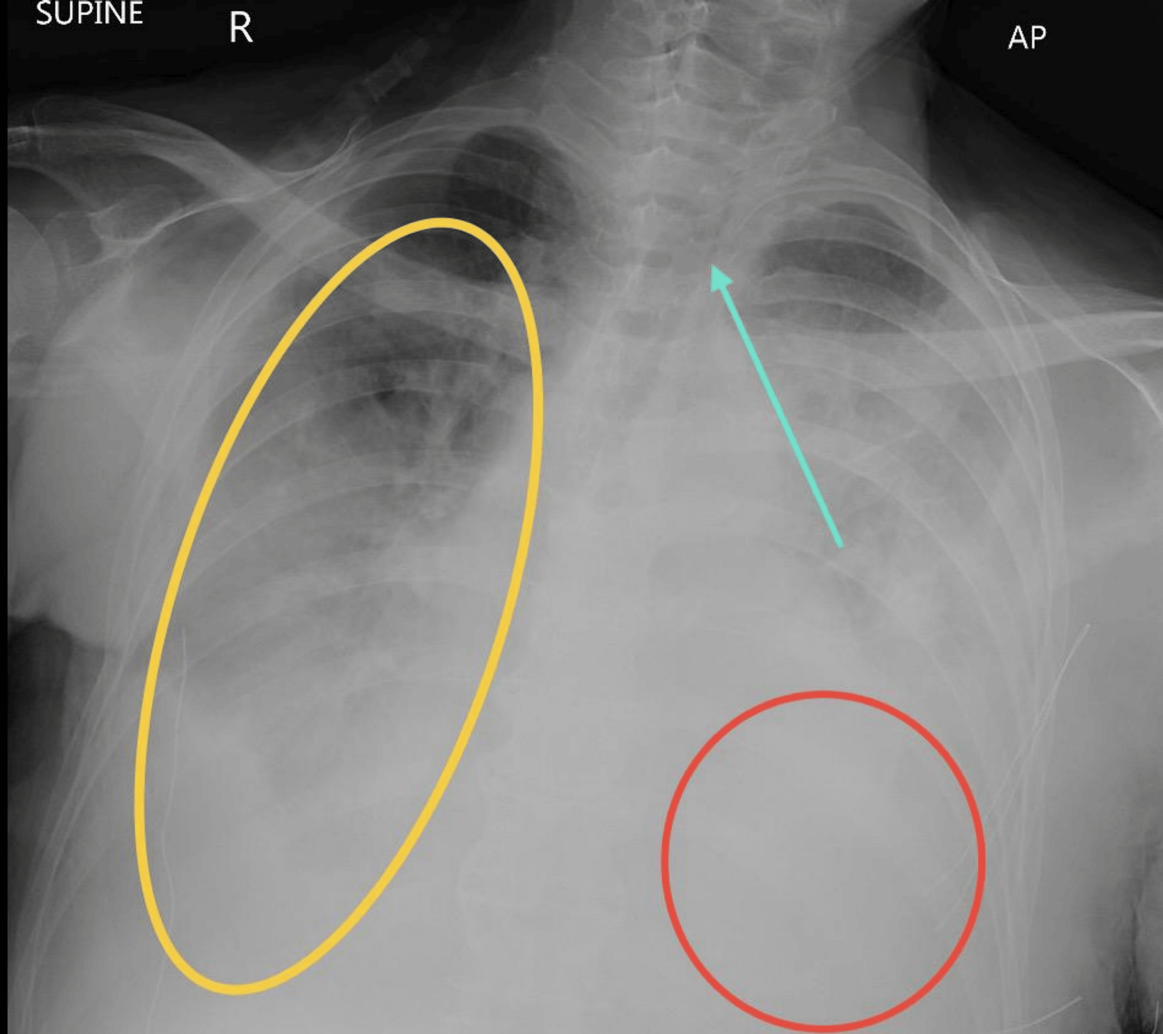
AP view of chest X-ray showing recurrent right-sided pleural effusion The patient is in the supine position. This AP view of the chest X-ray demonstrates opacification of the right hemithorax with a significant mediastinal shift to the left (blue arrow), consistent with a recurrent right-sided pleural effusion (yellow circle). The left lung shows patchy opacities (red circle). AP: anteroposterior

Following a month later, in late February 2025, she returned to the emergency department with worsening dyspnea associated with productive cough, brownish sputum, and poor oxygen intake on room air. A chest X-ray was done, which indicated severe left-sided pleural effusion with mediastinal shift to the right (Figure 5). She was admitted for pleural effusion drainage, and 800 ml was drained. She then developed recurrent right-sided pleural effusion, with leftward mediastinal shift (Figure 6). Wound and sputum cultures grew carbapenem-resistant *Pseudomonas aeruginosa*. Therefore, she was initiated on IV imipenem/relebactam.

By the end of March 2025, she showed gradual clinical improvement. She was hemodynamically stable, afebrile, and weaned off oxygen. Supportive care was continued with daily dressing, deep vein thrombosis and GI prophylaxis, and physiotherapy for mobilization, with recommendations for close outpatient follow-up. However, her symptoms started again, associated with altered sensorium and increased dyspnea. Arterial blood gas showed CO₂ retention, and there was rapid progression of right-sided effusion again. The patient was admitted to the ICU for monitoring of her complications. However, she went into bradyasystole. CPR with advanced cardiovascular life support protocol was initiated, but spontaneous circulation was not restored. She was pronounced dead and passed away in April 2025.

## Discussion

CM from breast cancer is relatively uncommon, with reported incidences ranging from 0.7% to 10.4% among all carcinomas [4]. CM presents on the chest wall, often as firm, flesh-colored to red nodules, papules, or plaques [5]. While CM can occur at any stage, its presence often indicates advanced disease and is associated with a poor prognosis [4]. TNBC, characterized by the absence of estrogen receptor, progesterone receptor, and HER2 expression, is known for its aggressive behavior and limited treatment options. Metaplastic breast carcinoma (MBC) is a subtype of TNBC that is particularly rare, comprising less than 1% of all breast cancers [6]. It is also histologically diverse, with squamous differentiation being one of its subtypes. This variant is notable for its aggressive clinical course and resistance to conventional therapies [7].

The development of CM with squamous features in TNBC is exceptionally rare. Squamous differentiation in the MBC subtype of TNBC is associated with a higher grade and poorer prognosis than other subtypes [7]. Clinically, these lesions can mimic benign dermatological conditions, leading to potential misdiagnosis and delayed treatment. Therefore, a high index of suspicion and prompt biopsy of suspicious skin lesions are crucial, especially in patients with a history of breast cancer. Routine skin checks should be performed during oncologic visits. Patients should also be educated on self-monitoring for new skin changes such as nodules, rashes, or any erythematous lesions, as this can help with prompt diagnosis. Management of CM in the context of MBC with squamous differentiation remains challenging due to its rarity and lack of standardized treatment protocols. Systemic chemotherapy is often employed; however, MBC frequently resists conventional chemotherapeutic agents [7]. Surgical excision and radiation therapy may be considered in select cases, but their efficacy is limited in widespread disease. Prognostically, the presence of CM signifies a poor outcome. Studies have shown that the median survival after the diagnosis of CM from breast cancer is approximately 13.8 months, with half of the patients succumbing within the first six months [4]. This highlights the aggressive nature of the disease and the need for early detection and intervention. Further research is also required to study the possible association of the disease with genetics.

## Conclusions

CM with squamous differentiation is a rare and aggressive presentation of TNBC, as highlighted by our case presentation. Skin lesions often appear within months to a few years of initial diagnosis and frequently coincide with visceral metastasis (lung, lymph nodes). Although the prognosis remains poor, it is essential to recognize the metastasis early to initiate appropriate management.
